# Point defect formation in M_2_AlC (M = Zr,Cr) MAX phases and their tendency to disorder and amorphize

**DOI:** 10.1038/s41598-017-10273-6

**Published:** 2017-08-29

**Authors:** S. H. Shah, P. D. Bristowe

**Affiliations:** Department of Materials Science and Metallurgy, University of Cambridge, Cambridge, CB3 0FS UK

## Abstract

First principles calculations are performed on Zr_2_AlC and Cr_2_AlC MAX phases to compare their ability to accommodate point defects under irradiation. Interatomic bonding is stronger in Cr_2_AlC than Zr_2_AlC but contrary to expectation Zr_2_AlC exhibits higher vacancy and antisite pair formation energies. However, interstitials and Frenkel defects are generally more difficult to form in Cr_2_AlC. The results are attributed to the mixed covalent/ionic/metallic nature of the bonding. Detailed comparison of all the energies suggests that the preferred defects in Zr_2_AlC and Cr_2_AlC are the V_Al_+Al_i_ Frenkel and Cr_Al_+Al_Cr_ antisite respectively. Thus the potential response of the two phases to irradiation is different and taking account of other competing defects it is suggested that Zr_2_AlC is less susceptible to amorphization.

## Introduction

A group of nanolaminated hexagonal materials called MAX phases have come under intense scrutiny in recent years due to their unusual physical properties that result from a combination of metallic and ceramic bonding characteristics^1^. They have the general formula M_n+1_AX_n_ where n is 1, 2 or 3, M is an early transition metal, A is an A-group element and X is either C or N. Among the properties that have drawn attention are their relatively high stiffness, good machinability, high thermal and electrical conductivity and good resistance to corrosion, oxidation, creep, fracture and fatigue^2^. Ti_3_SiC_2_ was one of the first MAX phases to be discovered and since then more than 70 others have been synthesized. Various applications for these materials have been suggested or put into practice including surface coatings, heating elements, bearings and armor.

The mixed covalent/ionic/metallic bonding present in MAX phases results in another possible application as an in-core structural material or coating within the hostile environment of a nuclear reactor^3, 4^. Under irradiation these phases have shown a remarkable ability to accommodate point defects and remain crystalline rather than becoming amorphous even when subjected to high levels of radiation or ion bombardment. However, sustained structural stability depends on the choice of M, A and X elements and some MAX phases are more irradiation tolerant than others. Furthermore, the elements should not have a high neutron cross-section since this will reduce the “permeability” of the material to neutrons and thus lower the reactor’s performance. Also a high neutron cross-section would, in some cases, cause undesirable activation products to form. Ti and Cr have relatively high neutron cross-sections (6.1 and 3.1 barn respectively) while Zr and Al are relatively low (0.184 and 0.233 barn). This suggests that Zr_n+1_AlC_n_ MAX phases might be candidates for nuclear applications.

Both Zr_2_AlC^5^ and Zr_3_AC_2_^6, 7^ have been recently synthesized although ZrC always appears as a secondary phase and under certain processing conditions Zr_2_AlC and Zr_3_AlC_2_ can exist together. Furthermore related quaternary compositions of the type Zr_2_(Al, A^I^)C where A^I^ = Sn, Sb, Pb or Bi have also been reported^8–11^. The latter study has suggested that partial substitutions on the A-site help to stabilize the Zr_2_AlC phase. However, partial substitutions on the M-site have only been successful for (Nb_x_,Zr_1-x_)_4_AlC_3_^12^. The structural stability and irradiation tolerance of Zr_n+1_AlC_n_ MAX phases can be assessed using density functional theory (DFT) calculations that focus on their formation enthalpy, bond strengths and propensity to form point defects^13^. If antisite point defect formation is relatively easy then the structure is more likely to remain crystalline than become amorphous since antisite defects retain the coherency of the lattice and act as a recovery mechanism. It is emphasized, however, that DFT calculations of defect energetics can only provide an indicator rather than a complete predictor of the susceptibility of a material to amorphize under irradiation since other process features such as kinetics are not taken into account. In this study we perform DFT calculations on Zr_2_AlC and compare its properties with Cr_2_AlC. Chromium is chosen as an alternative M element since it should enhance the high temperature oxidation resistance of the material^14^ and has a lower neutron cross-section (3.1barn) than Ti. In addition Cr_2_AlC has been successfully synthesized and the main radiation induced defects have been determined^15, 16^. Depending on the processing conditions, small amounts of Cr_3_C_2_, Cr_7_C_3_ and Cr_5_Al_8_ can be present in the material^17^ although generally speaking Cr_2_AlC has been synthesized (at the moment) with higher purity than Zr_2_AlC.

Previous computational studies^18^ using DFT have been performed on the phase stability and bulk properties of (Zr_1-x_ Cr_x_)_2_AlC including Zr_2_AlC and Cr_2_AlC. The calculations show that (Zr_1-x_ Cr_x_)_2_AlC is unstable with respect to dissociation into the Zr_2_AlC and Cr_2_AlC ternary phases and that this is due to the position of the Fermi level which lies at a peak position on the electronic density of states of (Zr_0.5_Cr_0.5_)_2_AlC. Attempts to synthesize such quaternary phases^11^ failed confirming the instability of these potential solid solutions. Point defect calculations have only been performed on Cr_2_AlC where it is found that the Cr-Al antisite pair has the lowest formation energy of all possible point defect combinations thus providing a defect mechanism for recovery during irradiation^19^. This result, as well as the finding that the C Frenkel pair also has relatively low formation energy, is consistent with the recent observations on ion irradiated Cr_2_AlC^15, 16^. Although the calculations were based on summing the energies of the individual defects they showed that despite the low magnetic moment of Cr, a non-magnetic treatment of Cr_2_AlC is sufficient if only trends in defect formation behavior are required. Furthermore, for the nuclear applications of interest here, the materials are synthesized and used at temperatures significantly above the observed Curie temperature (~73 K)^20^.

## Computational Method

The DFT calculations were performed using the projector augmented-wave (PAW) method^21^ as implemented in the VASP code^22, 23^ with the following states treated as valence: Zr (4s4p5s4d), Cr (3p4s3d), Al (3s3p), C (2s2p). Exchange correlation effects were included using the Generalized Gradient Approximation (GGA) as parameterized by Perdew-Burke-Ernzerhof (PBE)^24^. A Γ-point based Monkhorst-Pack scheme^25^ was used to sample the Brillouin zone with meshes sizes of 23 × 23 × 7 and 7 × 7 × 7 for perfect unit cells and supercells with defects respectively. A plane wave cutoff energy of 400 eV was used. Since all the MAX phases have metallic character, a Methfessel-Paxton smearing method with a sigma value of 0.2 was applied. In a fully optimized MAX phase structure the forces on each atom were always less than 0.01 eV/Å. Following the conclusions of previous work on Cr-containing systems^19^, the structures were treated as non-magnetic.

**Figure 1:**
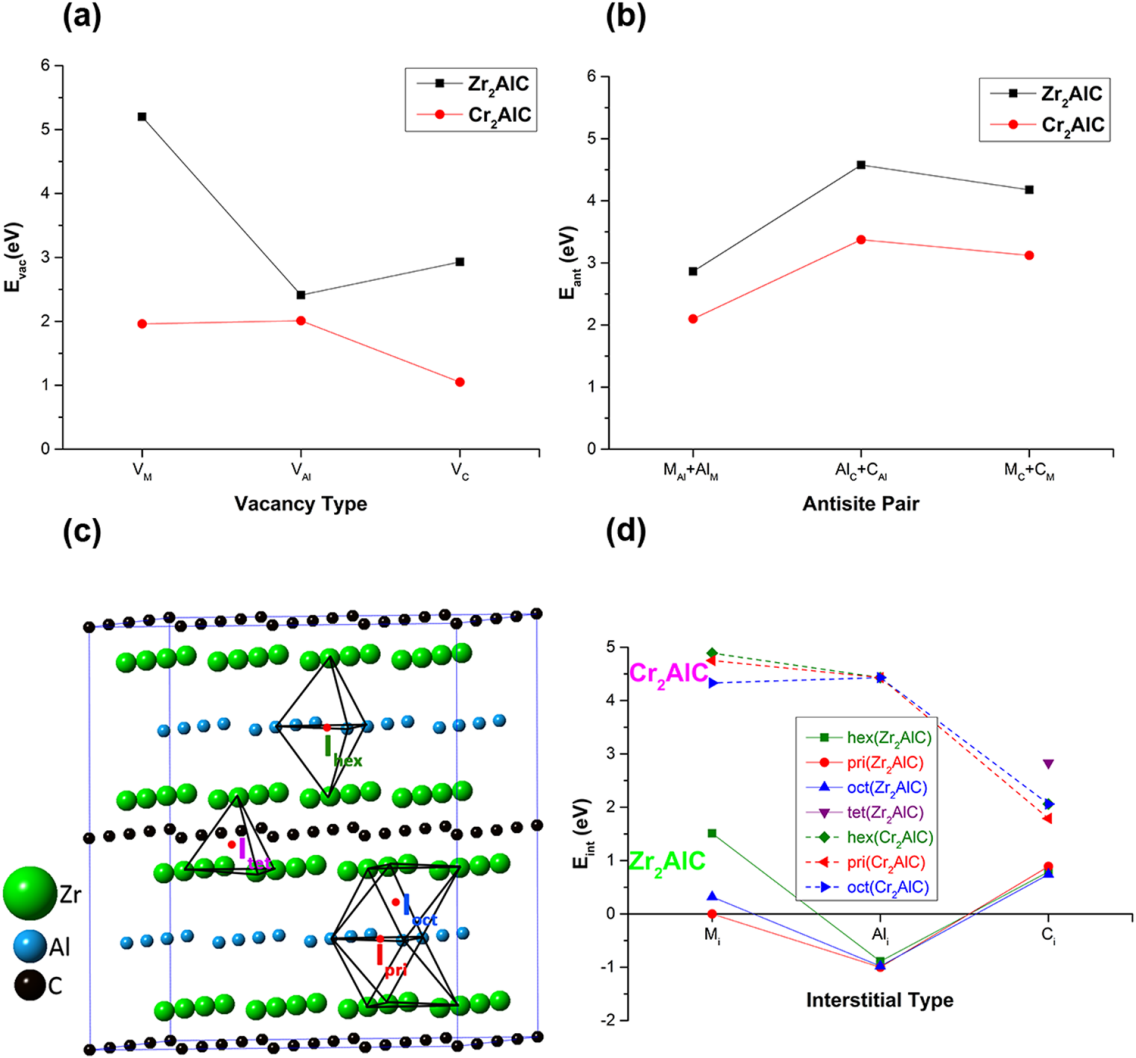
Formation energies (E_defect_) of different point defects in Zr_2_AlC and Cr_2_AlC. Here M is Zr/Cr. (**a**) vacancy defects, (**b**) antisite pair defects, (**c**) different interstitial configurations (I), (**d**) interstitial defects. For the vacancies and interstitials the chemical potentials of the pure constituent elements are used. The effect of varying the chemical potential is shown in Figures S3–S6. The antisite pair formation energies are independent of chemical potential. Numerical values are given in Table S3.

For the point defect calculations the supercell size used was 4 × 4 × 1 in order to minimize intercellular defect interactions. In both Zr_2_AlC and Cr_2_AlC, all cationic and anionic vacancies and interstitials were considered together with their associated Frenkel and antisite pairs. Since MAX phases are low-density layered structures, there is ample space between and within the layers for the incorporation of interstitial atoms. We have chosen four such open spaces and labeled them as I_hex_, I_tet_, I_oct_ and I_pri._ The I_hex_ position is in the Al layer and at the center of two triangular pyramids that connect to adjacent M layers. The I_tet_ position is at the center of a tetrahedron that connects adjacent M layers and crosses a C layer, and the I_oct_ position is at the center of an octahedron that connects an M layer to an adjacent Al layer. The I_pri_ position lies at the center of a triangular face of this octahedron within the Al layer. Each of the four symmetry distinct interstitial positions is shown in Fig. 1(c). For Frenkel pairs, the interstitials and vacancies are separated by at least 6 Å in order to avoid spontaneous annihilation of the vacancy-interstitial defect. For antisite pairs, atoms in neighboring layers are interchanged.

The procedure for determining point defect formation energies from supercell calculations is well established, see e.g. ref. 26, and requires knowledge of the chemical potentials of the constituent elements and the application of equilibrium conditions which prevent the formation of competing phases. The method has recently been applied to Ti-based MAX phases^13^. By calculating the formation enthalpies of the competing phases and applying the equilibrium conditions, the accessible range of chemical potentials required for the synthesis of Zr_2_AlC and Cr_2_AlC can be determined (see SI for details) and the general procedure has recently been automated^27^. The results are shown in Figures S1 and S2. For single point defects (vacancies or interstitials) the choice of chemical potential can significantly affect the formation energies as described below. However, for defect pairs (Frenkels or antisites), which are commonly found in irradiated compounds, the formation energies are independent of the chemical potentials.

To understand how the formation of point defects influences local bonding and hence structural stability we have performed a charge density analysis of bulk Zr_2_AlC and Cr_2_AlC with and without the defects. In particular Bader charges (i.e. atomic charges based on charge density) have been calculated together with the charge densities at the bond critical points (bcp) using QTAIMAC (Quantum Theory of Atoms in Molecules and Crystals)^28, 29^ as implemented in the Critic2 code^30^. The method is described in more detail in the SI.

## Results and Discussion

### Bulk Properties

**Figure 2:**
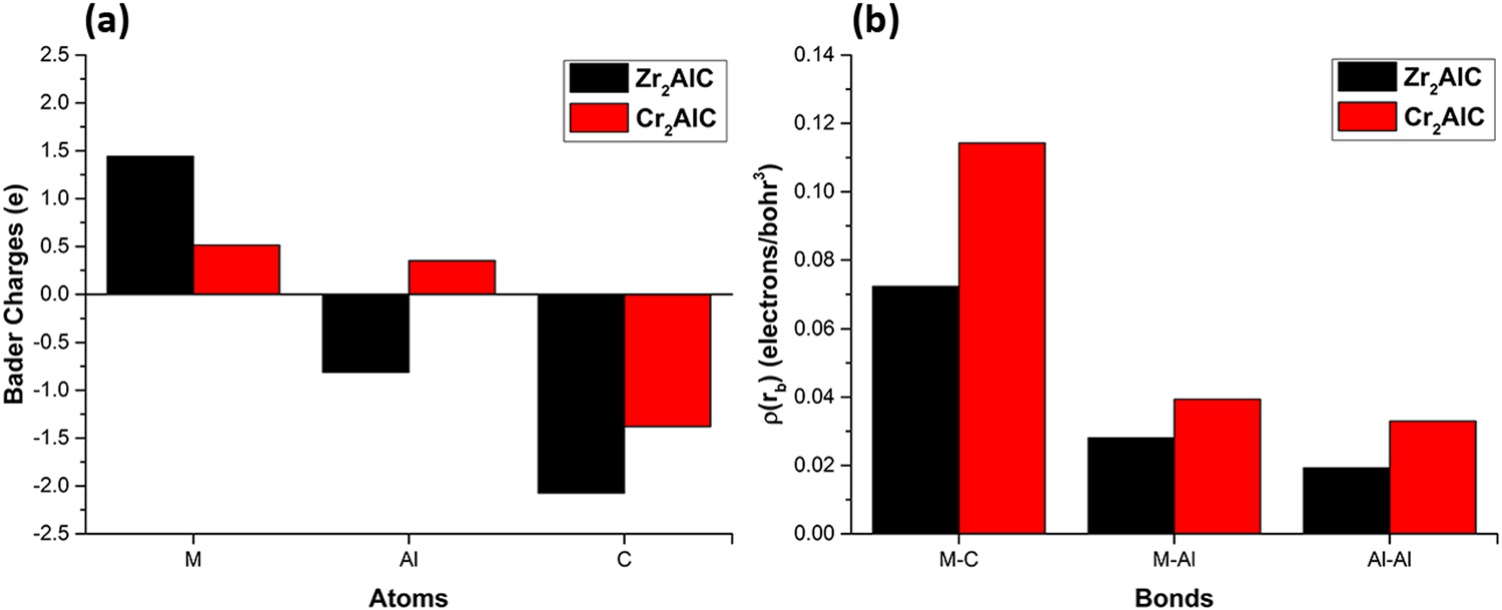
Charge density analysis of Zr_2_AlC and Cr_2_AlC. (**a**) Bader charges. (**b**) The charge density value at the bond critical point (bcp) between two bonded atoms. Numerical values are given in Table S2.

The calculated lattice parameters of Zr_2_AlC and Cr_2_AlC are shown in Table S1 and compared to experimental values. It is seen that the maximum deviation for Zr_2_AlC is only 0.16%. The relatively larger deviation for Cr_2_AlC (−1.12%) is due to the nonmagnetic state considered in this study. The Zr-containing phase has larger lattice parameters than the Cr-containing phase principally because of the larger crystal radius of Zr (13%). Figure 2 shows the calculated Bader charges on different constituent ions and the values of the charge densities at the bond critical points (bcp). Interestingly there is significant charge transfer from Zr to Al and C ions in Zr_2_AlC whereas charge transfer from Cr and Al to C in Cr_2_AlC is relatively small. Figure 2(b) clearly suggests that bonding in Cr_2_AlC is generally stronger than in Zr_2_AlC.

### Defect Formation

#### Vacancies

We investigated three types of vacancy (i.e.,V_Zr_/V_Cr_, V_Al_ and V_C_) in both Zr_2_AlC and Cr_2_AlC. It is clear from the calculated vacancy formation energies displayed in Fig. 1(a), that vacancies in Zr_2_AlC are much harder to create than in Cr_2_AlC and will therefore be less abundant. The values shown were determined using the chemical potentials of the constituent elements and, for Cr_2_AlC, agree well with the previous work^16^. The easiest vacancy to form in Cr_2_AlC is V_C_ whereas in Zr_2_AlC it is V_Al_. The Cr and Al vacancy formation energies in Cr_2_AlC are approximately equal. The larger vacancy formation energies in Zr_2_AlC are contrary to expectations based on the relative strengths of the bonds suggested by the bcp charge densities (Fig. 2(b)) and are due to the mixed and complex nature of the bonding as discussed in section 3.3.

The relative vacancy formation energies in Zr_2_AlC are different from Cr_2_AlC and can be summarized as follows:

$$\begin{array}{c}{{\rm{E}}}_{{\rm{vac}}}({\rm{Zr}}) > {{\rm{E}}}_{{\rm{vac}}}({\rm{C}}) > {{\rm{E}}}_{{\rm{vac}}}({\rm{Al}})\quad \,({{\rm{Zr}}}_{{\rm{2}}}{\rm{AlC}})\\ {{\rm{E}}}_{{\rm{vac}}}({\rm{Al}}) > {{\rm{E}}}_{{\rm{vac}}}({\rm{Cr}}) > {{\rm{E}}}_{{\rm{v}}{\rm{a}}{\rm{c}}}({\rm{C}})\quad ({{\rm{Cr}}}_{{\rm{2}}}{\rm{AlC}})\end{array}$$

Other MAX phases such as Ti_3_AlC_2_ and Ti_3_SiC_2_ exhibit a trend in vacancy formation similar to Zr_2_AlC^13, 31^. However, the results clearly depend on the synthesis conditions, as shown in the SI (Figures S3–S6). Figures S3 and S4 present the formation energies at the extreme points on the valid range of chemical potentials whereas Figures S5 and S6 show how these energies vary over the entire valid range. The latter two figures demonstrate that the energies behave uniformly between the extreme points and do not exhibit any local minima or maxima. Taken together several conclusions can be drawn from Figures S3–S6. In Zr_2_AlC, for example, under Zr-rich conditions, the vacancy trend becomes E_vac_(Zr) > E_vac_(Al) > E_vac_(C). It is noteworthy that vacancy formation energies depend strongly on the choice of chemical potentials (i.e., on the synthesis conditions). Under certain synthesis conditions, the vacancy formation energies for some of the vacancies (e.g., V_Al_, V_C_) in Zr_2_AlC become smaller than the corresponding vacancies in Cr_2_AlC as shown in Figures S3 and S4. The Zr and C vacancies are the least and most affected vacancies in Zr_2_AlC respectively due to the chemical potentials. The Zr vacancy remains the most difficult vacancy to form in Zr_2_AlC irrespective of the choice of chemical potential. Nonetheless, V_Zr_ becomes relatively easier to form under Al rich conditions. Al vacancies compared to carbon vacancies become relatively harder to form in Zr_2_AlC under Zr and/or Al rich conditions. The most significant effect of the Zr_2_AlC synthesis environment is on the formation of C vacancies. V_C_ becomes the most stable vacancy under Zr and/or Al rich conditions. Consequently depending on how Zr_2_AlC is synthesized, either V_Al_ or V_C_ becomes the most stable vacancy. This is different to the situation in Cr_2_AlC where the choice of chemical potential has a negligible effect on the vacancy formation energies. For instance, C vacancies remain the most stable vacancies under all the synthesis conditions.

#### Antisite pairs

The antisite pair energies give an indication of how easy it is to create disorder on the different MAX phase sublattices. They also provide a recovery mechanism for the crystal following an irradiation induced displacement cascade. This mechanism depends on the type of the interstitial/vacancy species and the target sublattice. The antisite defect formation energies are shown in Fig. 1(b) for Zr_2_AlC and Cr_2_AlC. Unlike single vacancies (or interstitials) the energies do not depend on the chemical environment since the atom species simply interchange and there is no reservoir needed for the deposition or extraction of atoms.

Figure 1(b) shows that antisite pairs are harder to create in Zr_2_AlC than in Cr_2_AlC and that of the three antisite combinations, M_Al_+Al_M_ pairs are the most stable. The results can be understood in simple terms using differences in the crystal radii^32^ and electronegativities^33^ of the atoms involved. For example, the percentage difference in crystal radius between M and Al is less than between M and C or Al and C (R_Zr_ = 0.86 Å, R_Cr_ = 0.76 Å, R_Al_ = 0.53 Å, R_C_ = 0.29 Å). Similarly the percentage difference in electronegativity between M and Al is less than between M and C or Al and C (χ_Zr_ = 1.33, χ_Cr_ = 1.66, χ_Al_ = 1.61, χ_C_ = 2.55). Both differences suggest that M_Al_+Al_M_ pairs should be the preferred antisite defects and indicate that elastic and electronic effects are playing a role. Comparing Zr_Al_+Al_Zr_ with Cr_Al_+Al_Cr_ it is seen that the percentage difference in both quantities is greater in Zr_2_AlC (e.g. 62% versus 43% for the crystal radii and 21% versus 3% for the electronegativities) explaining the larger formation energy of the Zr_Al_+Al_Zr_ defect. Interchanging the cations (Zr/Cr and Al) with the anions (C) is always relatively difficult. In this case the carbon atom in the cation layer eventually moves into an interstitial position between a Zr/Cr layer and Al layer as shown in Figures S7 and S8. Similarly the cations (Zr/Cr and Al) also do not like to be in a carbon layer and create a vacancy by moving into the next available cation layer.

#### Interstitials

MAX phases are low-density layered structures with ample open space between and within the layers. When these phases are irradiated a large number of atoms are displaced from their equilibrium sites and form interstitials. The energy required to form these interstitials varies depending on the chemistry of MAX phase and the type of interstitial atom and interstice. We have investigated the formation of three different types of interstitial (Zr_i_/Cr_i_, Al_i_, C_i_) in four different interstices (I_hex_, I_pri_, I_oct_ and I_tet_, see Fig. 1(c)) in bulk Zr_2_AlC and Cr_2_AlC. The interstitial atoms at the I_tet_ site are found to be either unstable or have relatively large formation energies in both Zr_2_AlC and Cr_2_AlC. This is most likely due to the limited space available for Zr/Cr and Al, which are cations with large radii. As a consequence, we now focus only on the salient features of the different interstitials at the sites I_hex_, I_pri_ and I_oct_.

The calculated interstitial formation energies are displayed in Fig. 1(d) where it is seen that interstitials are much harder to create in Cr_2_AlC and will therefore be relatively less abundant. The values shown were determined using the chemical potentials of the constituent elements and, for Cr_2_AlC, agree well with the previous work^16^. For Cr_2_AlC it is also seen that there is little difference in formation energy between the different sites for a given interstitial suggesting that they may have relaxed to similar configurations and that the C interstitial has the lowest energy. For Zr_2_AlC, it is clear that the Al interstitial has the lowest energy, which is negative, and apparently the same in all sites. The negative formation energy suggests that Zr_2_AlC can spontaneously become super-stoichiometric in Al and there is some experimental evidence for an excess of Al in some Ti-based MAX phases^34^. However, as noted in our discussion of vacancy formation energies, the results will depend on the synthesis conditions and Figures S3 and S5 shows that the Al interstitial formation energy becomes almost positive when the thermodynamically valid range of chemical potentials is considered. The trend in behavior, however, does not change (i.e. Al_i_ remains the most favorable interstitial). Interestingly under some synthesis conditions (i.e., values of chemical potential), certain interstitials (e.g., C interstitials) become harder to form in Zr_2_AlC than in Cr_2_AlC (see Figures S3/S4 and S5/S6). In Zr_2_AlC, Zr/Al and C interstitials are the least and most affected by the chemical potentials respectively. Like Al interstitials in Zr_2_AlC, Zr interstitials remain mostly unaffected by the synthesis conditions except in Al rich conditions that make them relatively harder to form. The most pronounced effect of the chemical potentials is on C interstitials in Zr_2_AlC. They become harder to form under Zr and/or Al rich conditions. Nevertheless, the Al interstitial remains the most stable interstitial in Zr_2_AlC irrespective of the chemical environment. On the other hand, the chemical potentials only slightly influence the different interstitial types in Cr_2_AlC as seen in Figure S6 by the relatively small variation in colors representing the formation energies. The C interstitial remains the most stable interstitial in Cr_2_AlC under all possible synthesis conditions that stabilize pure Cr_2_AlC. Cr and Al interstitials become relatively the most difficult to form depending on the synthesis environment. For instance, formation of a particular interstitial (e.g., Cr_i_) becomes harder if any other species (e.g., Al and/or C) is in rich condition.

The almost overlapping energies in Fig. 1(d) suggest that the relaxed configurations should be examined in detail. Figure S9 shows these configurations viewed parallel to the relevant Al layer in the initial structure. We first consider the M(=Zr/Cr) interstitial which is seen to remain in or close to the adjacent Al layer. Interestingly, starting at the I_pri_ position, these interstitials occupy a lattice site in the Al layer and create an Al interstitial. In the case of Zr_2_AlC, this lowers the energy considerably, making the process very favorable. The M interstitials in the I_oct_ position initially are relatively unstable. For Zr_2_AlC, the Zr atom relaxes into the Al plane whereas for Cr_2_AlC, the Cr atom stands off from the Al plane. We now consider the Al and C interstitials, which exhibit a particular behavior in Zr_2_AlC and Cr_2_AlC, namely, their formation is almost site independent and they always adjust their position in or near the adjacent Al layer. For Zr_2_AlC, all the Al interstitials spontaneously relax into the Al layer, lowering the energy of the structure considerably as noted above. The same configurations are formed in Cr_2_AlC but at much higher cost in energy. The difference in behavior can be explained, at least qualitatively, by considering the differences in local structure. Figure S10 compares the Al bond lengths and M-Al layer spacings in Zr_2_AlC and Cr_2_AlC with bulk fcc Al. Compared to the bulk metal, Zr_2_AlC is in tension whereas Cr_2_AlC is in compression. Thus the formation of an Al interstitial in Zr_2_AlC is more likely to be favorable than in Cr_2_AlC.

The C interstitial behaves differently in Zr_2_AlC and Cr_2_AlC. For Zr_2_AlC, the C interstitial in the Al layer remains in the Al layer, as in the case of the I_hex_ and I_pri_ sites. The C interstitial starting at the I_oct_ site comes close to the Al layer but does not settle in this layer. However, for Cr_2_AlC, the C interstitial moves from the I_hex_ site to a position in between the Cr and Al layers, as does the C interstitial starting at the I_oct_ site. On the other hand the C interstitial at I_pri_ remains in the Al layer. Energetically the most favorable site in Zr_2_AlC for Zr and Al interstitials is I_pri_ and for C interstitials is I_oct_. For Cr_2_AlC, I_oct_ is the most favorable site for the Cr interstitial and I_pri_ for the C interstitial. The Al interstitials in Cr_2_AlC are position independent.

#### Frenkel pairs

**Figure 3:**
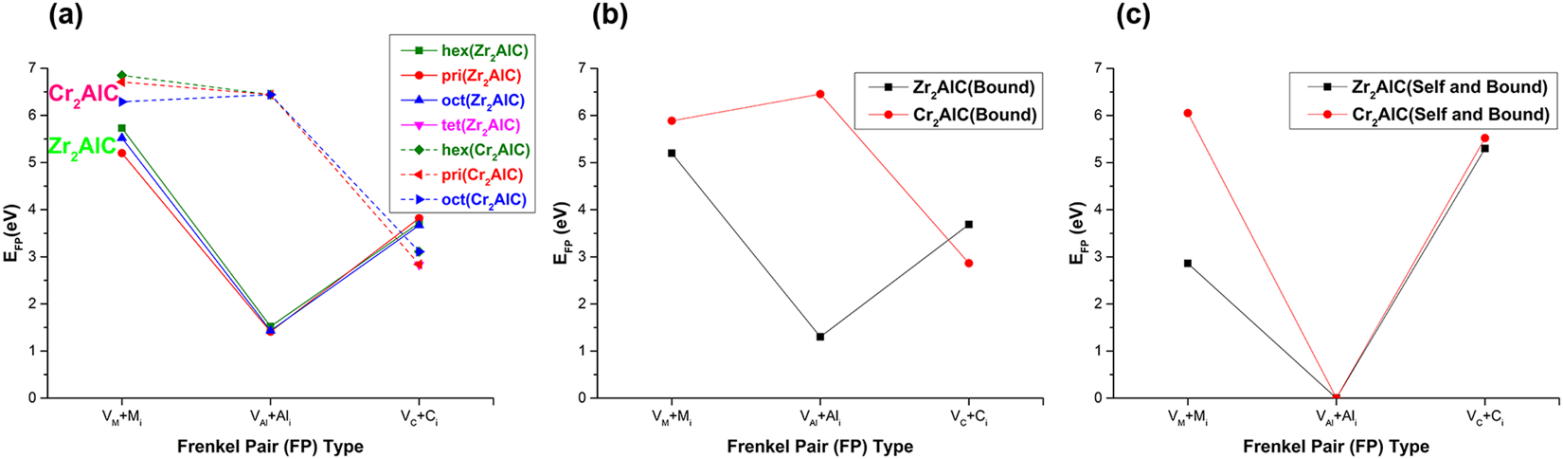
The formation energies of different types of Frenkel pair defects in Zr_2_AlC and Cr_2_AlC. Here M is Zr/Cr. (**a**) Isolated Frenkel pairs (**b**) bound Frenkel pairs (**c**) self-Frenkel pairs. The Frenkel pair formation energies are independent of chemical potential. Numerical values are given in Table S3.

Frenkel pairs are some of the most commonly found defects in irradiated materials. An atom is displaced off its lattice site to form a vacancy and an interstitial. We have determined Frenkel pair formation energies in Zr_2_AlC and Cr_2_AlC in three different ways. Firstly, we have considered *isolated* Frenkel pairs whose energy is determined simply by summing the formation energies of the corresponding vacancy and interstitial under the same chemical conditions. We note that like antisite defects, the Frenkel formation energies should be independent of the chemical potentials (provided they are the same for the vacancy and interstitial) since no reservoir is needed. This has been the method used in previous defect calculations on MAX phases, e.g. ref. 19. Secondly, we have considered *bound* Frenkel pairs in which the vacancy and interstitial are relaxed within the same computational cell and initially 5–9 Å apart across M, Al or C layers. In order to keep the number of computations feasible, we have considered only those Frenkel pairs that have the lowest isolated formation energies. Lastly, we have considered a special type of bound Frenkel pair in which vacancy and interstitial are created in the same layer and call them *self*-Frenkel pairs. Figure 3 compares the formation energies obtained using the three methods.

It is seen that the form and magnitude of the isolated (Fig. 3(a)) and bound (Fig. 3(b)) Frenkel pair formation energies are very similar indicating that the pair binding energy is very small and that bringing the two point defects closer together does not change the preferred configuration, i.e. V_Al_+Al_i_ in Zr_2_AlC and V_C_+C_i_ in Cr_2_AlC. It is also seen that M and Al Frenkel pairs are more difficult to form in Cr_2_AlC than in Zr_2_AlC whereas C Frenkel pairs are somewhat easier to form in Cr_2_AlC than in Zr_2_AlC. This is because M and Al interstitials in Cr_2_AlC are very difficult to create whereas in Zr_2_AlC they could be energetically favorable. The relative stability of C and Al Frenkel pairs is also found in other MAX phases. For instance, C Frenkel pairs are found to be stable in Ti_3_AlC_2_, Ti_2_AlN and Cr_2_GeC and Al Frenkel pairs are found to be stable in Ti_3_SiC and Ti_2_AlC^33^. The M type Frenkel pairs always require a large amount of energy to generate which is a consequence of the relatively strong M-C bonding. The relaxed structures of the bound Frenkel pairs are shown in Figures S11 and S12 for Zr_2_AlC and Cr_2_AlC respectively. It is seen that displacements away from the initial configurations are small, which reflects the small binding energies. The I_pri_ site is the preferred position of the most stable Frenkel pair in Zr_2_AlC (V_Al_+Al_i_) and in Cr_2_AlC (V_C_+C_i_).

Lastly, we consider the self-Frenkel pair formation energies that are shown in Fig. 3(c). It might be expected that a self-Frenkel pair created in its own layer would recombine on relaxation and thus cost no energy to form. This is what happens with the Al self-Frenkel pair in both Zr_2_AlC and Cr_2_AlC but more complex relaxations take place for the M and C self-Frenkel pairs. The displacements they create are shown in Figures S13 and S14. The formation energies of these defects are generally higher than their bound counterparts because secondary defects form. In Zr_2_AlC, the Zr self-Frenkel pair relaxes to form a Zr_Al_+Al_Zr_ antisite pair and the C self-Frenkel pair stimulates the formation of a Zr Frenkel pair. In Cr_2_AlC, both the Cr self-Frenkel pair and the C self-Frenkel pair remain but relax to a different orientation.

### Radiation Tolerance

In order to fully understand the irradiation tolerance of a material at the atomistic level, it is vital to investigate all possible recovery mechanisms that help it to recrystallize and resist amorphization. The above results indicate that the energy to form a vacancy or antisite defect in Zr_2_AlC is higher than in Cr_2_AlC. However, interstitials and Frenkel defects are generally more difficult to form in Cr_2_AlC. Comparison of all the energies suggests that the preferred defects in Zr_2_AlC and Cr_2_AlC are the V_Al_+Al_i_ Frenkel and Cr_Al_+Al_Cr_ antisite respectively. However, Zr_Al_+Al_Zr_ antisites and V_C_+C_i_ Frenkels are also energetically favorable in these materials and could compete or interact with the preferred defects during irradiation and subsequent cooling. Thus the potential response of the two phases to irradiation is different. Structurally, Zr_2_AlC tends to form defects that retain the coherency of the lattice (e.g. Figure S11(d) for the V_Al_+Al_i_ Frenkel and Figure S7(b) for the Zr_Al_+Al_Zr_ antisite) while Cr_2_AlC tends to form a defect that disturbs coherency (e.g. Figure S12(f) for the V_C_+C_i_ Frenkel). Disturbance of the lattice suggests that Cr_2_AlC is more susceptible to amorphization. The equilibrium concentrations of defects at finite temperature are straightforward to calculate from Boltzmann statistics^35^. For example at 1100 K, the concentration of Cr_Al_+Al_Cr_ antisite pairs in Cr_2_AlC would exceed the concentration of Zr_Al_+Al_Zr_ antisite pairs in Zr_2_AlC by about a factor of 100. Similarly, at the same temperature, the concentration of V_Al_+Al_i_ Frenkel pairs in Zr_2_AlC would exceed the concentration of V_c_+C_i_ Frenkel pairs in Cr_2_AlC by about a factor of 2000. However, it is emphasized that these are equilibrium concentrations and likely to be less than those found *in situ* in a radiation environment.

**Figure 4:**
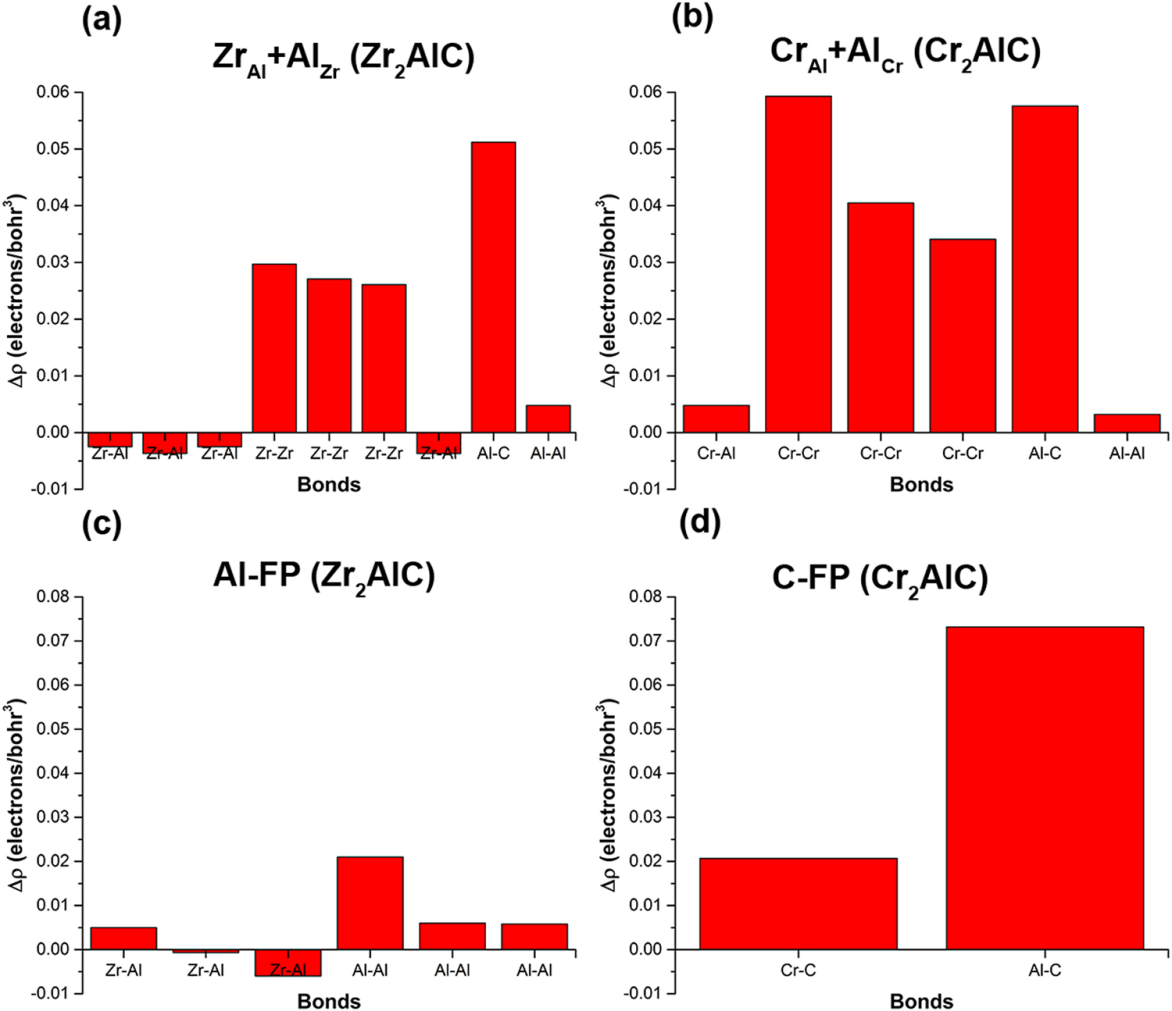
The change in the charge density at bond critical points due to a defect in Zr_2_AlC and Cr_2_AlC. (**a**) Zr_Al_+Al_Zr_ antisite pair (**b**) Cr_Al_+Al_Cr_ antisite pair (**c**) Al Frenkel pair (FP) (**d**) C Frenkel pair (FP).

To further understand how each of these defects affect the strength of the MAX phase we have determined how the charge densities at the bond critical points change using the QTAIMAC method^28–30^. Figure 2(b) clearly shows that all the bonds in Zr_2_AlC are weaker than the corresponding bonds in Cr_2_AlC. This is corroborated by another theoretical study^36^ which also finds that the bulk modulus of Cr_2_AlC is greater than Zr_2_AlC. Three types of bonds (M-C, M-Al, Al-Al) exist in bulk M_2_AlC (M = Zr/Cr) phases where M-C and Al-Al bonds are the strongest and weakest bonds respectively in both systems (Fig. 2(b)). Bonding in Zr_2_AlC is predominately ionic as indicated by the positive value of the Laplacian of the charge density whereas in Cr_2_AlC, the Cr-Al bond is found to be covalent (see Table S2). The comparatively weak ionic bonding in Zr_2_AlC renders it more radiation tolerant than Cr_2_AlC since defects more easily re-establish themselves with the crystal structure^13, 37^. It is interesting to note that bonding in several relevant Zr-based compounds (Zr-metal, Zr-carbides, Zr-aluminides) is found to be weaker than the bonding in Cr-based compounds. For instance, Figure S15 compares the strength of M-C, M-Al and Al-Al bonds in Zr_2_AlC, Cr_2_AlC and the relevant binaries and metallic systems. Almost all the bonds in Zr-based compounds are relatively weaker than the corresponding bonds in Cr-based compounds. This could imply that Zr-based materials intrinsically have more ability to re-establish defects with the initial crystal structure compared to Cr-based materials. Defects disturb the local bonding by breaking old bonds and creating new bonds. Figure 4 shows the change in bcp charge density (bond strength) due to a defect in Zr_2_AlC and Cr_2_AlC. For instance, in the case of cationic antisite M_Al_+Al_M_ (M = Zr/Cr) defect pairs two new strong bonds, namely M-M and Al-C, are formed in both systems. Most importantly all the bonds including old and newly formed due to defects are stronger in Cr_2_AlC than in Zr_2_AlC. Similarly, the most stable defects, namely, Al Frenkel pairs in Zr_2_AlC and C Frenkel pairs in Cr_2_AlC also show relatively stronger bonds in Cr_2_AlC compared to Zr_2_AlC. The relatively weak and ionic bonding in Zr_2_AlC makes it comparatively more radiation tolerant than Cr_2_AlC by ‘recrystallizing’ defects more easily with the initial crystal structure.

## Conclusions

Using DFT calculations combined with a chemical potential and charge density analysis, the relative stability of various point defects in two MAX phases, Zr_2_AlC and Cr_2_AlC, has been investigated. The objective has been to determine their relative tendency to disorder and amorphize under irradiation. It is found that interatomic bonding in Cr_2_AlC is generally stronger than in Zr_2_AlC but contrary to expectation Zr_2_AlC exhibits higher vacancy and antisite pair formation energies. Although both materials are metallic they exhibit different degrees of ionicity and covalency, with Zr_2_AlC being the more ionic. This difference affects the vacancy formation energies, which would otherwise be larger in the stronger metal. Nevertheless, interstitials and Frenkel defects are generally more difficult to form in Cr_2_AlC. Analysis of the defect formation energies within the accessible range of chemical potentials shows that their relative values do not change significantly compared to pure conditions, especially for Cr_2_AlC. In Zr_2_AlC the main effect is to increase the Al interstitial formation energy so that it becomes almost positive. Detailed comparison of all the energies suggests that the preferred defects in Zr_2_AlC and Cr_2_AlC are the V_Al_+Al_i_ Frenkel and Cr_Al_+Al_Cr_ antisite respectively. Although DFT calculations are only indicators rather than predictors of a material’s susceptibility to amorphise under irradiation, the current results suggest that that Zr_2_AlC is less susceptible to amorphization because the defects that form preserve the coherency of the lattice and offer a viable recovery mechanism.

## Electronic supplementary material

Supplementary Information
