# Supplementary material for: Point defect formation in M2AlC (M = Zr,Cr) MAX phases and their tendency to disorder and amorphize
Source: Sci Rep. 2017 Aug 29;7:9667. doi: 10.1038/s41598-017-10273-6 (PMC5574895; doi:10.1038/s41598-017-10273-6)
Supplement: Supplementary file 1 — Supplementary Information [file 41598_2017_10273_MOESM1_ESM.pdf]

Supplementary Information

Point defect formation in M_2AlC (M = Zr,Cr) MAX phases and their tendency to disorder and amorphize

S H Shah and P D Bristowe*
Department of Materials Science and Metallurgy
University of Cambridge, Cambridge CB3 0FS, U.K.
E-mail: pdb1000@cam.ac.uk

Table S1: Comparison of the experimental (Exp.) and calculated (Cal.) lattice parameters of Zr_2AlC and Cr_2AlC. Z_M is the z coordinate of the M(=Zr/Cr) atom.

	Zr_2AlC(Exp.)[1]	Zr_2AlC(Cal.)	%Dev.	Cr_2AlC(Exp.)[2]	Cr_2AlC(Cal.)	%Dev.
a=b(Å)	3.3237	3.3184	+0.16	2.8570	2.8339	-0.79
c (Å)	14.5705	14.5877	+0.12	12.81	12.6665	-1.12
V (Å^3)	139.40	139.11	-0.20	90.55	88.10	-2.68
Z_M	0.0871	0.0866	-0.57	0.086	0.085	-1.16

Table S2: Comparison of the calculated Bader charges, bcp charge densities and Laplacians in Zr_2AlC and Cr_2AlC.

Atoms	Zr_2AlC-Bader Charges (e)	Cr_2AlC-Bader Charges (e)
M	1.443	0.514
Al	-0.812	0.352
C	-2.074	-1.380

Bonds	Zr_2AlC-charge density at bcp (e/b^3)	Zr_2AlC-Laplacian of Charge density at bcp	Cr_2AlC-change density at bcp (e/b^3)	Cr_2AlC-Laplacian of Charge density at bcp
M-C	0.0723	+0.1451	0.1143	+0.2519
M-Al	0.0281	+0.0162	0.0394	-0.0075
Al-Al	0.0193	+0.0056	0.0330	+0.0042

Table S3: The calculated defect formation energies, E_{defect}(eV), in Zr_2AlC and Cr_2AlC compared with literature values. Chemically rich conditions are assumed for the vacancies and interstitials.

Defect Type	Notation	E_{defect} (eV)-Zr_2AlC	E_{defect}(eV)-Cr_2AlC	Others – Cr_2AlC
Vacancy				
	V_M	5.2	1.96	1.936[3]
	V_{Al}	2.41	2.01	2.090[3]
	V_C	2.93	1.05	0.976[3]
Antisite Pair				
	M_{Al}+Al_M	2.86	2.09	2.265[4],2.40[5]
	Al_C+C_{Al}	4.58	3.37	20.661[4],8.86[5]
	M_C+C_M	4.18	3.12	9.480[4],7.53[5]
Interstitials				
	M_i(hex)	1.51	4.89	
	Al_i(hex)	-0.89	4.43	
	C_i(hex)	0.79	2.06	
	M_i(pri)	0	4.75	
	Al_i(pri)	-1	4.43	
	C_i(pri)	0.89	1.79	
	M_i(oct)	0.32	4.33	4.526[3]
	Al_i(oct)	-0.98	4.43	5.226[3]
	C_i(oct)	0.74	2.06	2.192[3]
Frenkel Pair (Bound)				
	V_M+M_i	5.2	5.89	7.05[5]
	V_{Al}+Al_i	1.30	6.46	7.26[5]
	V_C+C_i	3.69	2.86	3.14[5]
Frenkel Pair (Self)				
	V_M+M_i	2.86	6.05	
	V_{Al}+Al_i	0	0	
	V_C+C_i	5.3	5.52	

Calculation of defect formation energies and the accessible range of chemical potentials

The formation energy E_{defect} of a charge neutral point defect d depends on the chemical potentials μ_i as follows:

$$E_{defect}(d) = [E_T(d) - E_T(0)] \pm \sum_i n_i \mu_i$$

where E_T(d) and E_T(0) are the total energies of the defective and perfect supercells respectively and n is the number of atoms of type i. Taking Zr_2AlC as an example, the following equilibrium conditions can be applied based on the observation that ZrC and Zr_3AlC_2 are phases that compete with Zr_2AlC [1]:

$$2\mu_{Zr} + \mu_{Al} + \mu_C = \Delta H_f(Zr_2AlC) \quad (1)$$

$$\mu_{Zr} + \mu_C \leq \Delta H_f(ZrC) \quad (2)$$

$$3\mu_{Zr} + \mu_{Al} + 2\mu_C \leq \Delta H_f(Zr_3AlC_2) \quad (3)$$

$$\mu_{Zr} \leq 0, \quad \mu_{Al} \leq 0, \quad \mu_C \leq 0 \quad (4)$$

where ΔH_f is the formation enthalpy of the relevant compound. Eqn. (1) defines the equilibrium condition and constrains the formation of primary material (Zr_2AlC). Eqns. (2) and (3) define constraints on the chemical potentials of the constituent elements to avoid the formation of ZrC and Zr_3AlC_2 (the competing secondary phases). Eqn. (4) restricts the formation of the constituent elements (Zr, Al, C). These are the four fundamental equations that define the formation of Zr_2AlC in favor of the constituent elements and/or the competing phases. In order to obtain the region of stability in chemical potential space, further manipulation of these equations is required. For instance, we may rewrite Eqn.(1) by expressing μ_{Zr} in terms of μ_{Al} and μ_C which then defines a two dimensional chemical potential space. Similarly, we can rewrite Eqns. (2) and (3) in terms of μ_{Al} and μ_C using Eqn. (1). Additionally the lower bounds on μ_{Zr}, μ_{Al} and μ_C are obtained by combining Eqns. (1) and (4). Once all the equations are formed, they have to be solved in combinations by employing LU (lower upper) decomposition and back-substitution methods. More details can be found in [7].

The analogous set of equations for Cr_2AlC are based on the observation that Cr_3C_2 and Cr_7C_3 are phases that compete with Cr_2AlC [6]:

$$2\mu_{Cr} + \mu_{Al} + \mu_C = \Delta H_f(Cr_2AlC)$$

$$3\mu_{Cr} + 2\mu_C \leq \Delta H_f(Cr_3C_2)$$

$$7\mu_{Cr} + 3\mu_C \leq \Delta H_f(Cr_7C_3)$$

$$\mu_{Cr} \leq 0, \quad \mu_{Al} \leq 0, \quad \mu_C \leq 0$$

By calculating the formation enthalpies and applying the equilibrium conditions, the valid range of chemical potentials required for the synthesis of Zr_2AlC and Cr_2AlC can be determined [7]. These are shown in Figure S1 and S2 and indicated by the coloured regions. The point defect formation energies can be determined using any valid set of chemical potentials ($\mu_{Zr/Cr}$, μ_{Al}, μ_C) that lie within these regions. Figure S3 and S4 focus only on the defect formation energies determined at the apices A, B, C, D and E, which represent the chemical extremes. Figure S5 and S6 show how these energies vary over the entire valid range of chemical potentials. It is seen that the formation energies behave uniformly between the extremes and do not exhibit any local minima or maxima within the valid range.

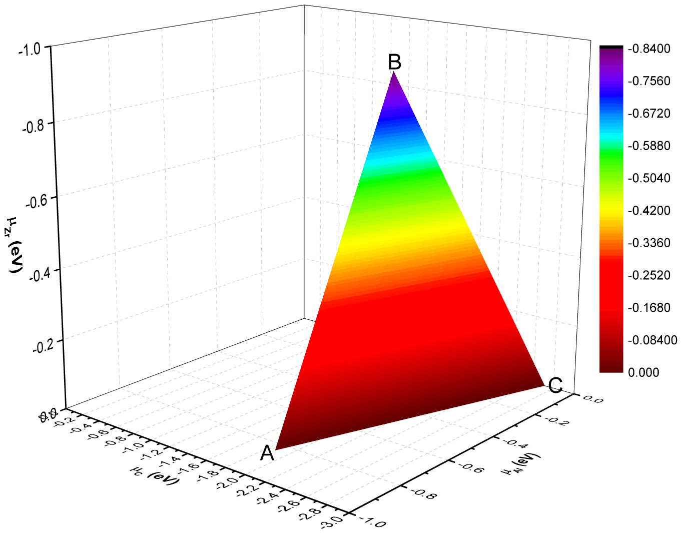

Figure S1: Chemical potential space (μ_{Zr} ,μ_C , μ_{Al}) showing the accessible range of chemical potentials (triangular region) that stabilize Zr_2AlC. Results obtained using the CPLAP code [7]. Colors within the triangle indicate the value of μ_{Zr}.

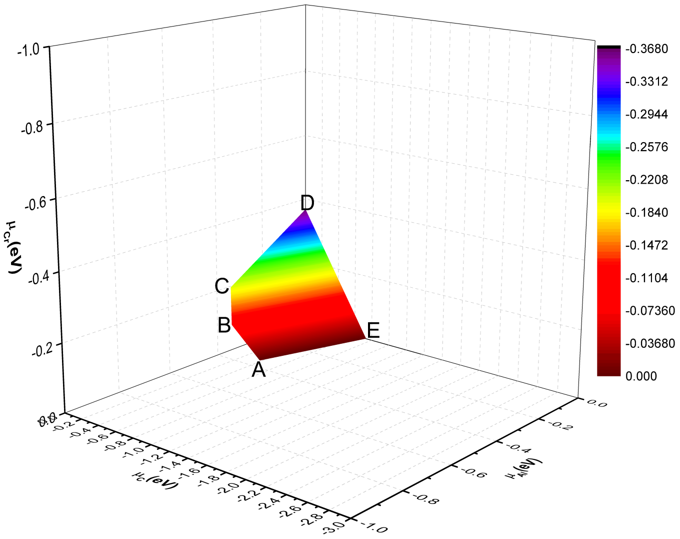

Figure S2: Chemical potential space (μ_{Cr} ,μ_C , μ_{Al}) showing the accessible range of chemical potentials (polygonal region) that stabilize Cr_2AlC. Results obtained using the CPLAP code [7]. Colors within the polygon indicate the value of μ_{Cr}. Note that the accessible range is much smaller compared to Zr_2AlC.

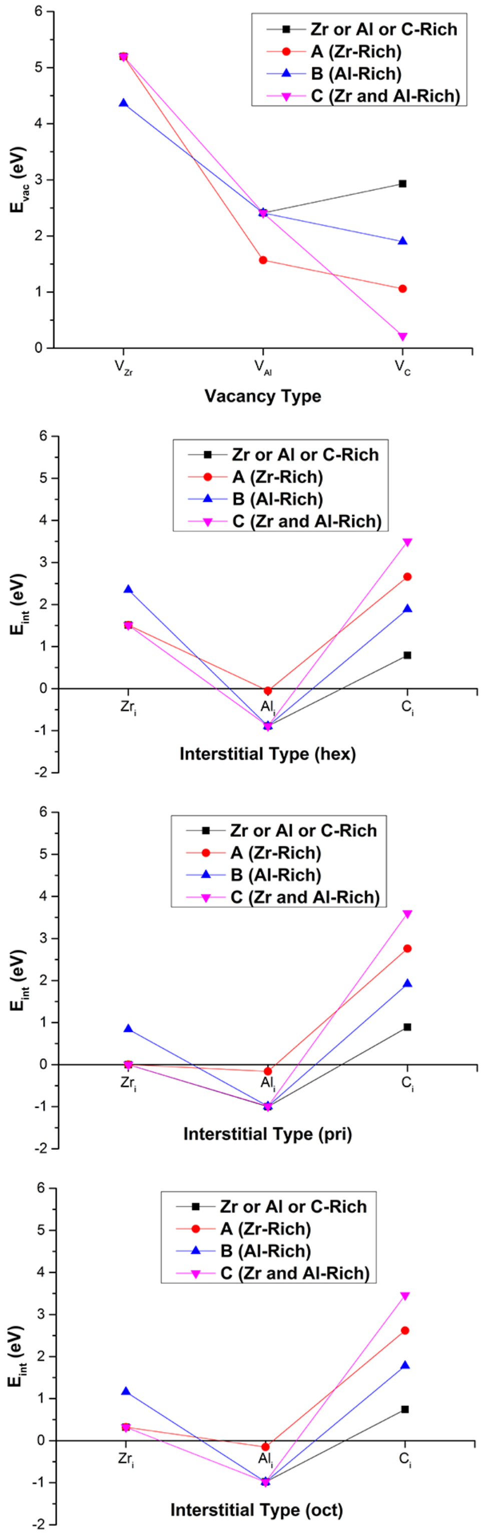

Figure S3: Defect formation energies (vacancies and interstitials) in Zr_2AlC computed under chemically rich conditions (Zr/Al/C Rich) and at points A, B and C defined by the apices of the colored region in Figure S1.

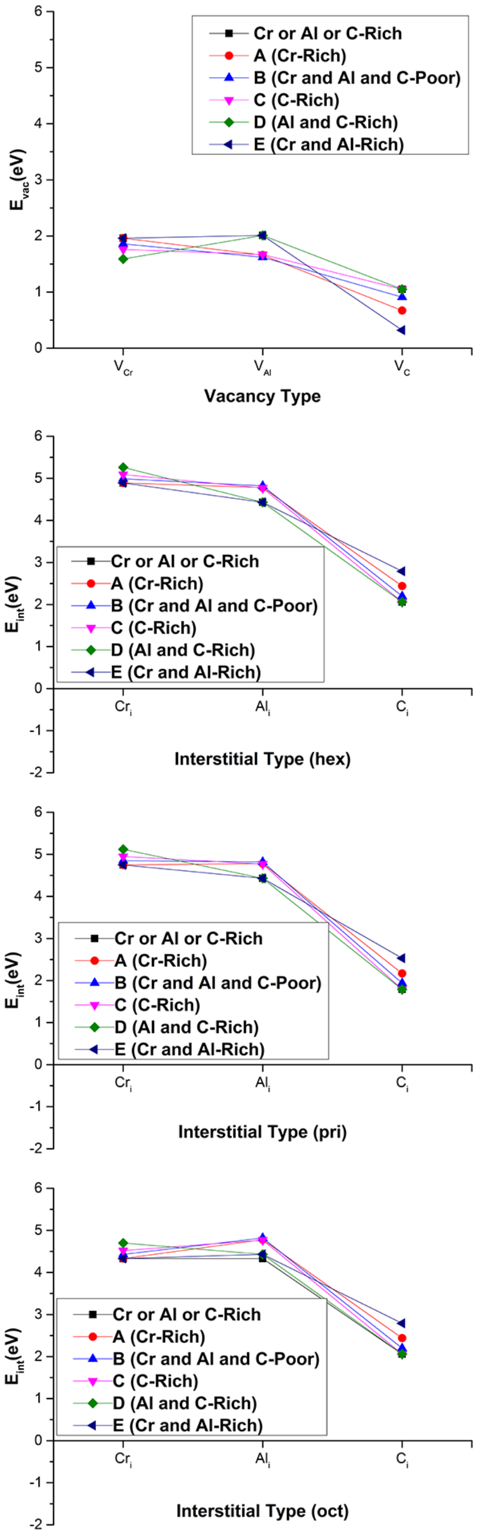

Figure S4: Defect formation energies (vacancies and interstitials) in Cr_2AlC computed under chemically rich conditions (Cr/Al/C Rich) and at points A, B, C, D and E defined by the apices of the colored region in Figure S2.

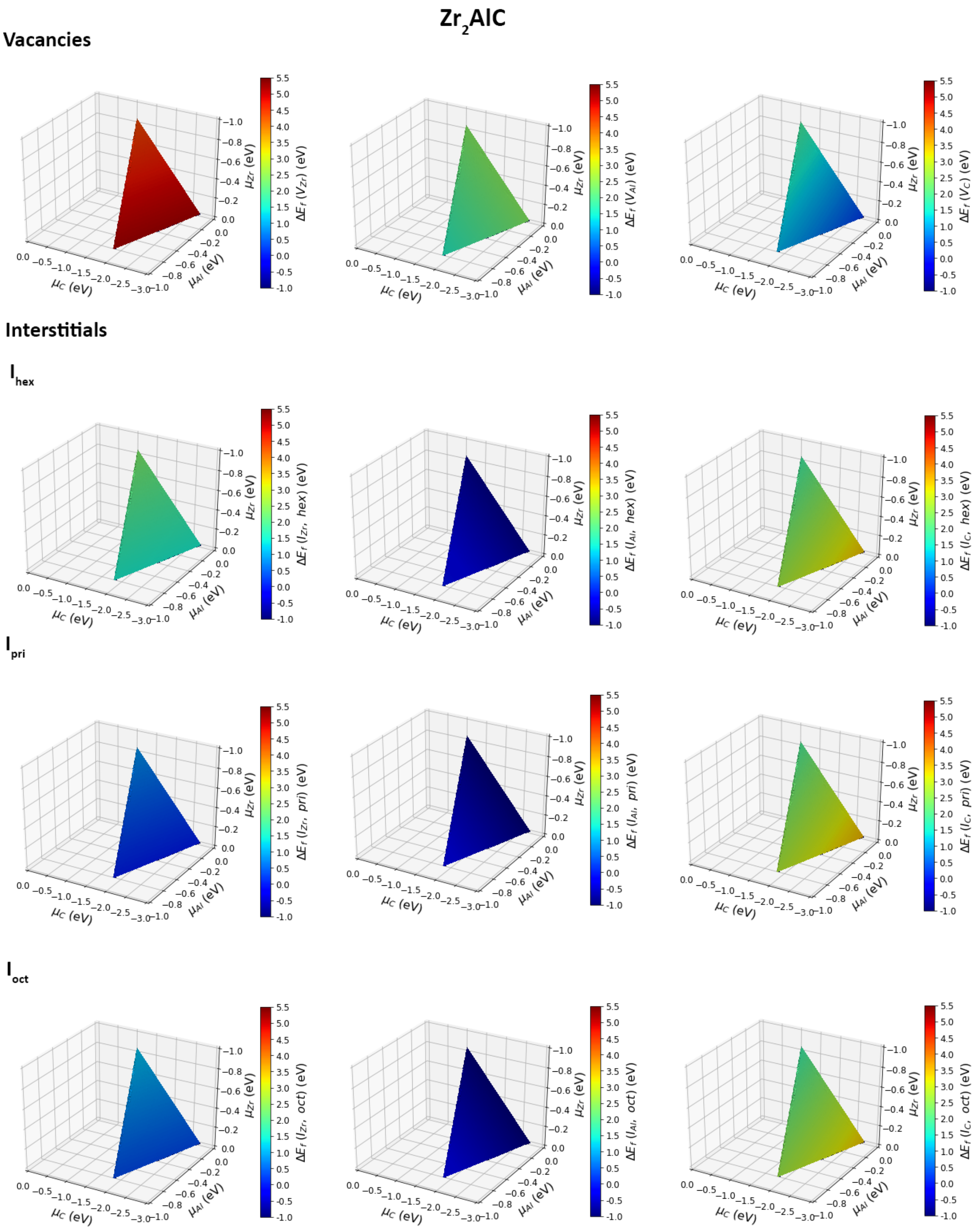

Figure S5: Defect formation energies (vacancies and interstitials) in Zr_2AlC over the entire accessible range of chemical potentials defined by the triangle ABC in Figure S1. Colors within the triangles indicate the formation energies.

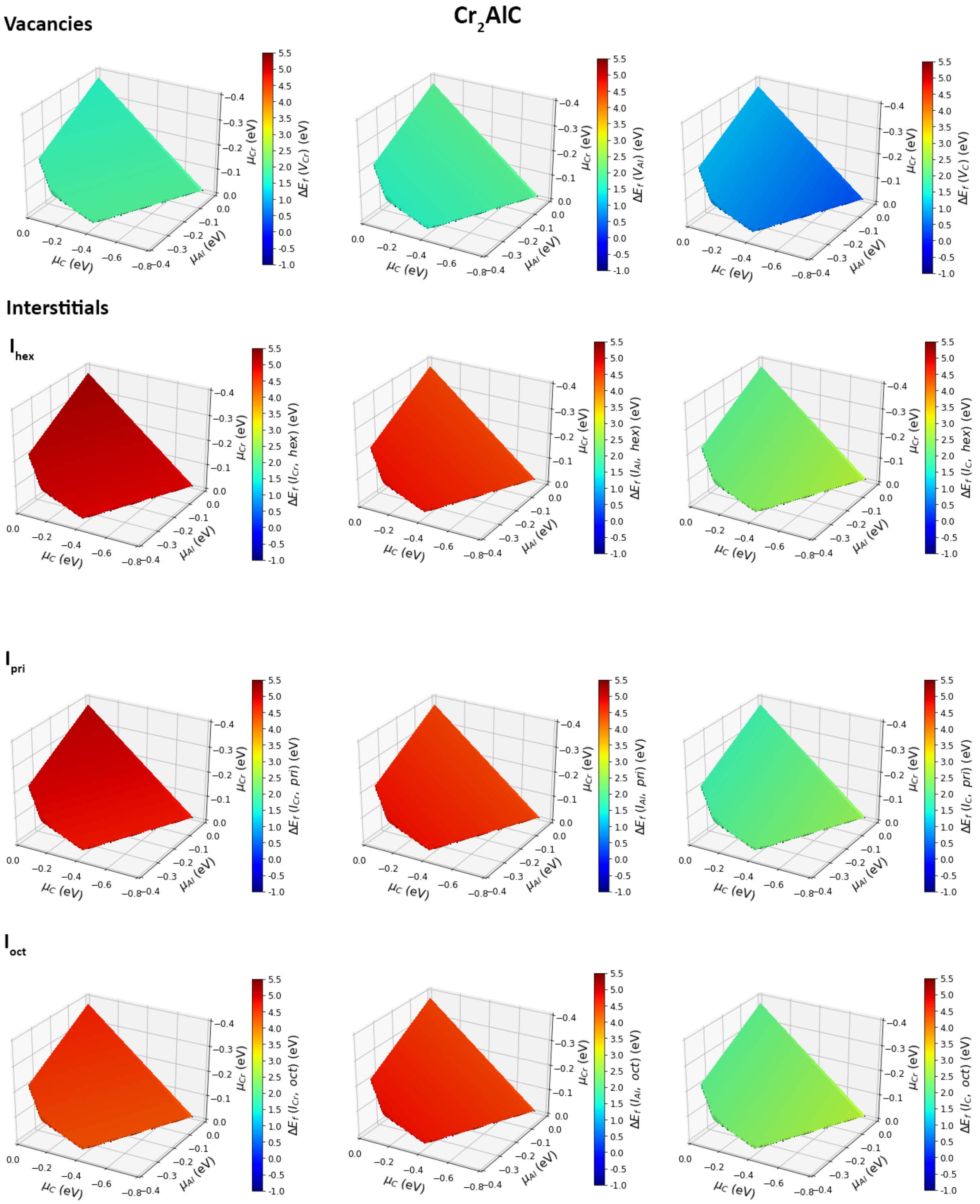

Figure S6: Defect formation energies (vacancies and interstitials) in Cr_2AlC over the entire accessible range of chemical potentials defined by the polygon ABCDE in Figure S2. Colors within the polygons indicate the formation energies.

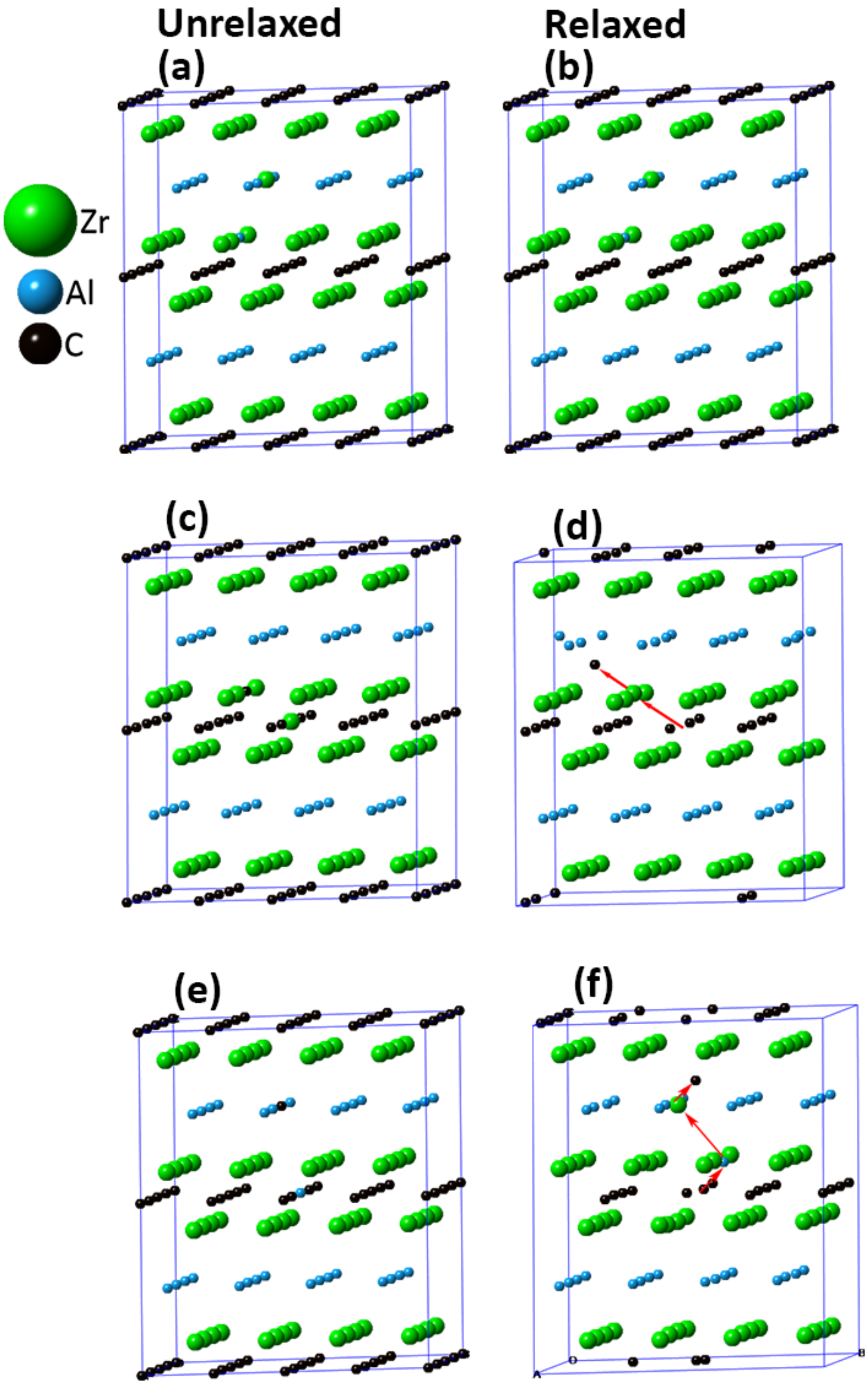

Figure S7: The unrelaxed and relaxed structures of Zr_2AlC with antisite pair defects (a) Unrelaxed Zr_{Al}+Al_{Zr} (b) Relaxed Zr_{Al}+Al_{Zr} (c) Unrelaxed Zr_C+C_{Zr} (d) Relaxed Zr_C+C_{Zr} (e) Unrelaxed Al_C+C_{Al} (f) Relaxed Al_C+C_{Al}. Arrows indicate how atoms are displaced during relaxation.

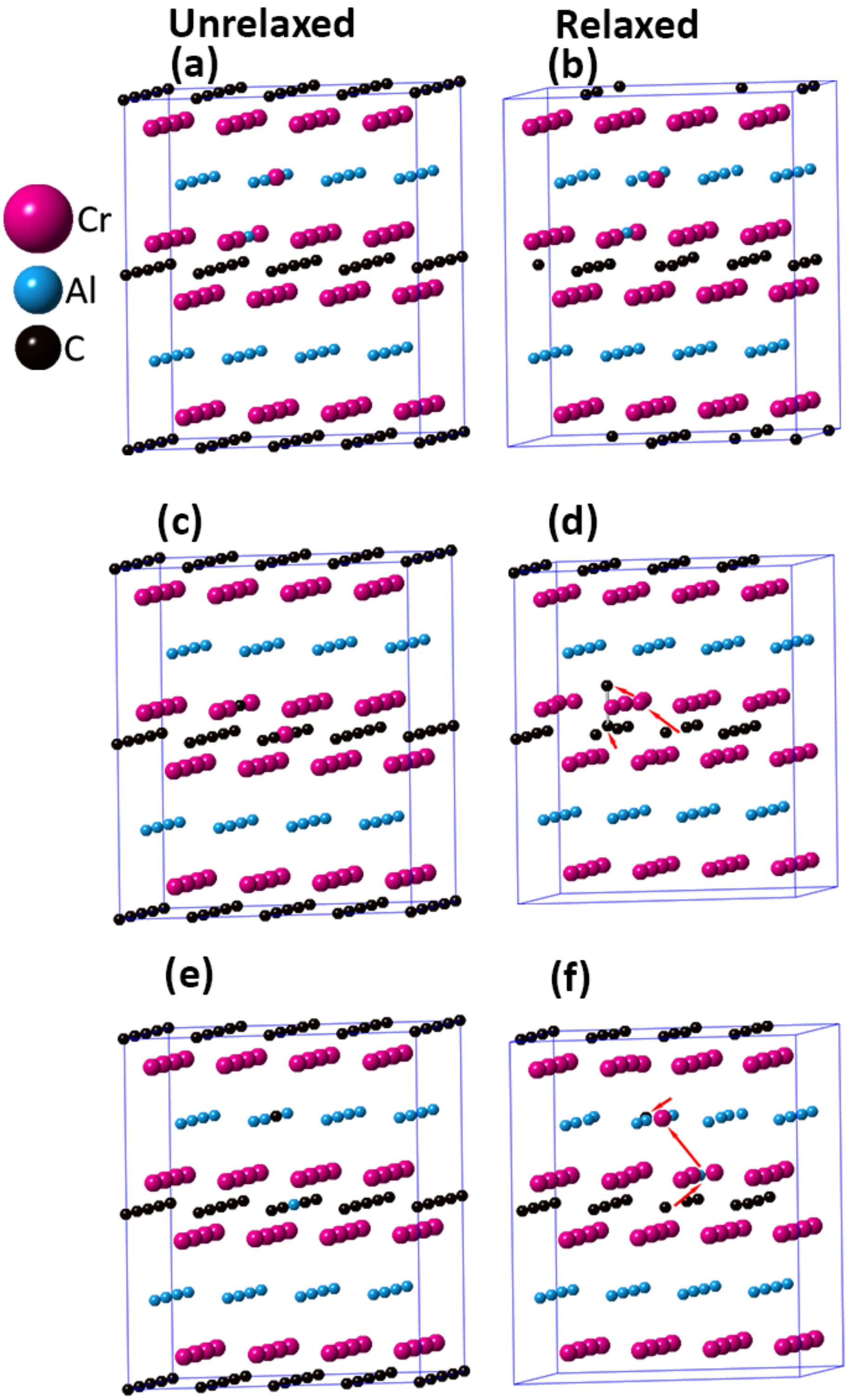

Figure S8: The unrelaxed and relaxed structures of Cr_2AlC with antisite pair defects (a) Unrelaxed Cr_{Al}+Al_{Cr} (b) Relaxed Cr_{Al}+Al_{Cr} (c) Unrelaxed Cr_C+C_{Cr} (d) Relaxed Cr_C+C_{Cr} (e) Unrelaxed Al_C+C_{Al} (f) Relaxed Al_C+C_{Al}. Arrows indicate how atoms are displaced during relaxation.

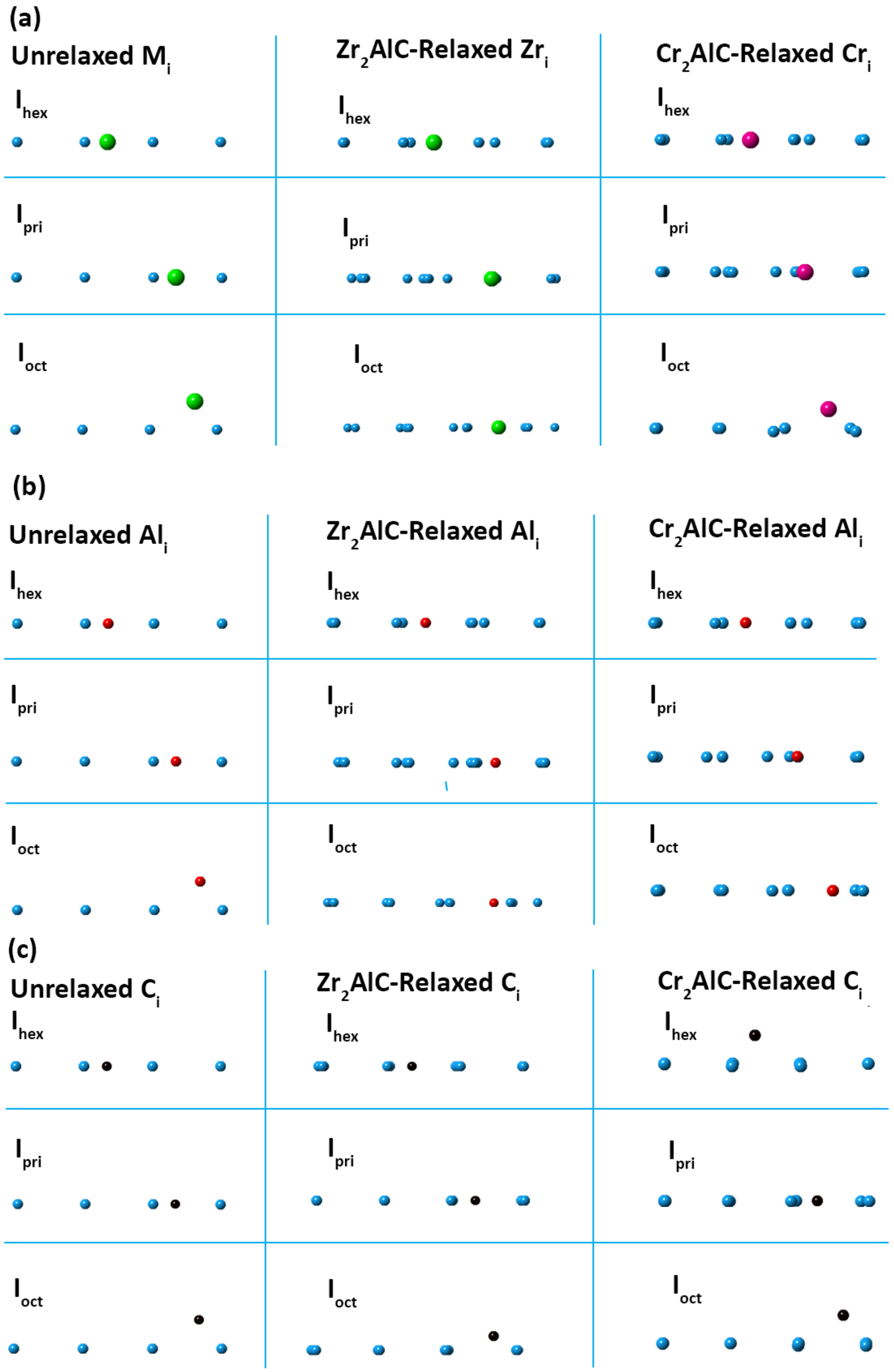

Figure S9: The unrelaxed and relaxed structure of different interstitials (I) in Zr_2AlC and Cr_2AlC. Only the relevant Al layer and the interstitial are shown. (a) M_i (=Zr_i/Cr_i) interstitials (b) Al_i interstitials. The Al interstitial is shown in red to distinguish it from the other atoms in the Al layer. (c) C_i interstitials

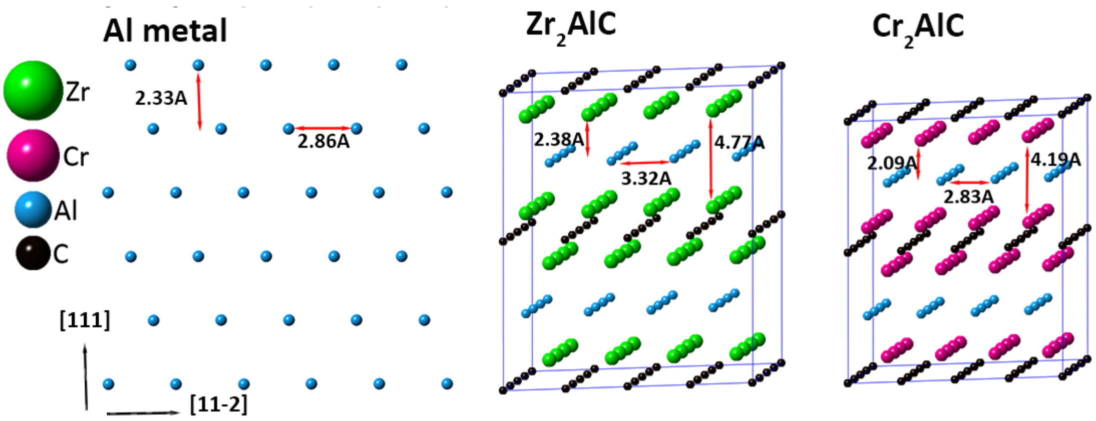

Figure S10: Comparison of the bond lengths and inter-planar spacings in fcc Al, Zr_2AlC and Cr_2AlC.

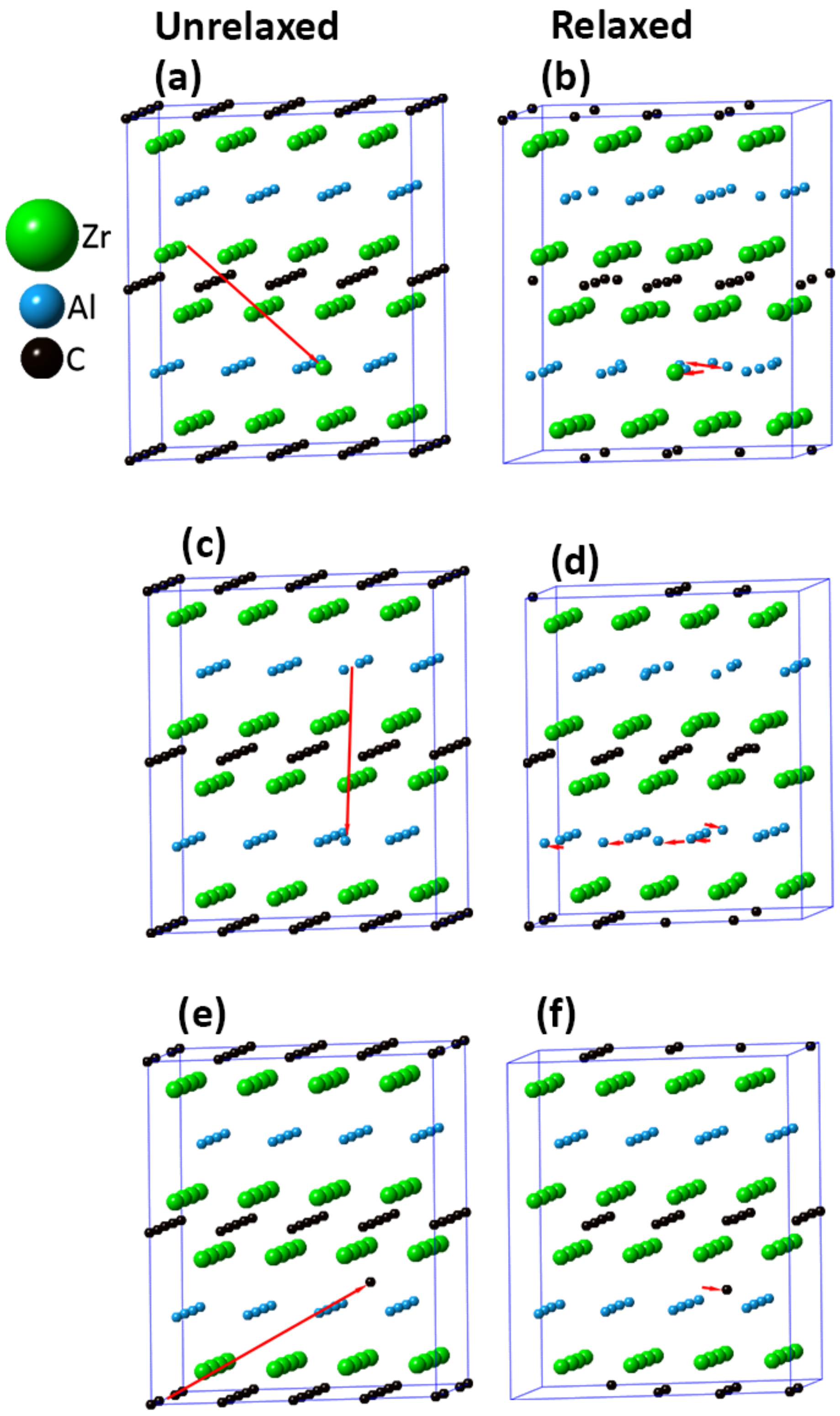

Figure S11: The unrelaxed and relaxed structures of Zr_2AlC with different types of bound Frenkel pairs. (a) Unrelaxed V_{Zr}+Zr_i (b) Relaxed V_{Zr}+Zr_i (c) Unrelaxed V_{Al}+ Al_i (d) Relaxed V_{Al}+Al_i (e) Unrelaxed V_C+C_i (f) Relaxed V_C+C_i. Arrows indicate how atoms are displaced initially and then during relaxation.

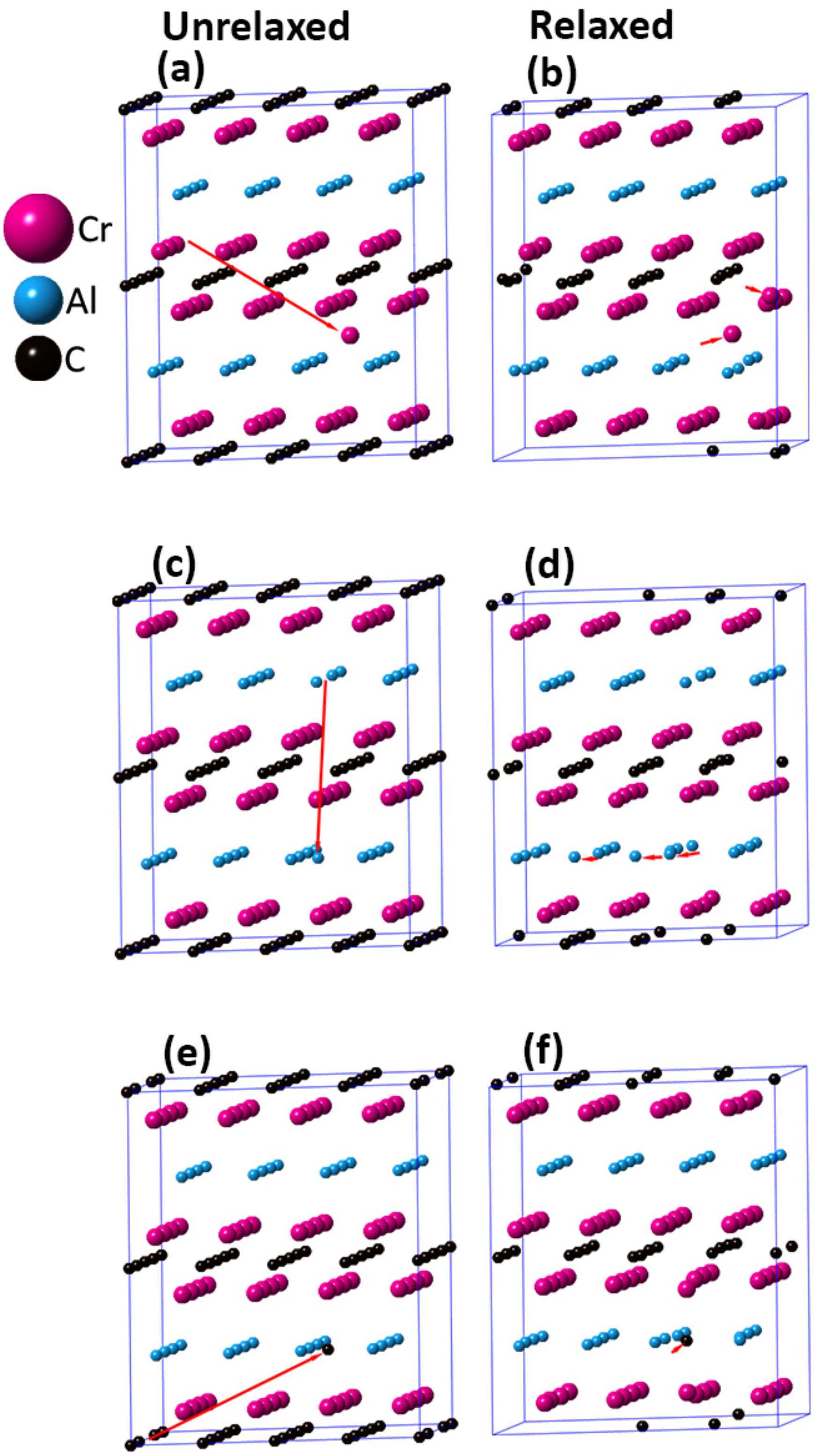

Figure S12: The unrelaxed and relaxed structures of Cr_2AlC with different types of bound Frenkel pairs. (a) Unrelaxed V_{Cr}+Cr_i (b) Relaxed V_{Cr}+Cr_i (c) Unrelaxed V_{Al}+Al_i (d) Relaxed V_{Al}+Al_i (e) Unrelaxed V_C+C_i (f) Relaxed V_C+C_i. Arrows indicate how atoms are displaced initially and then during relaxation.

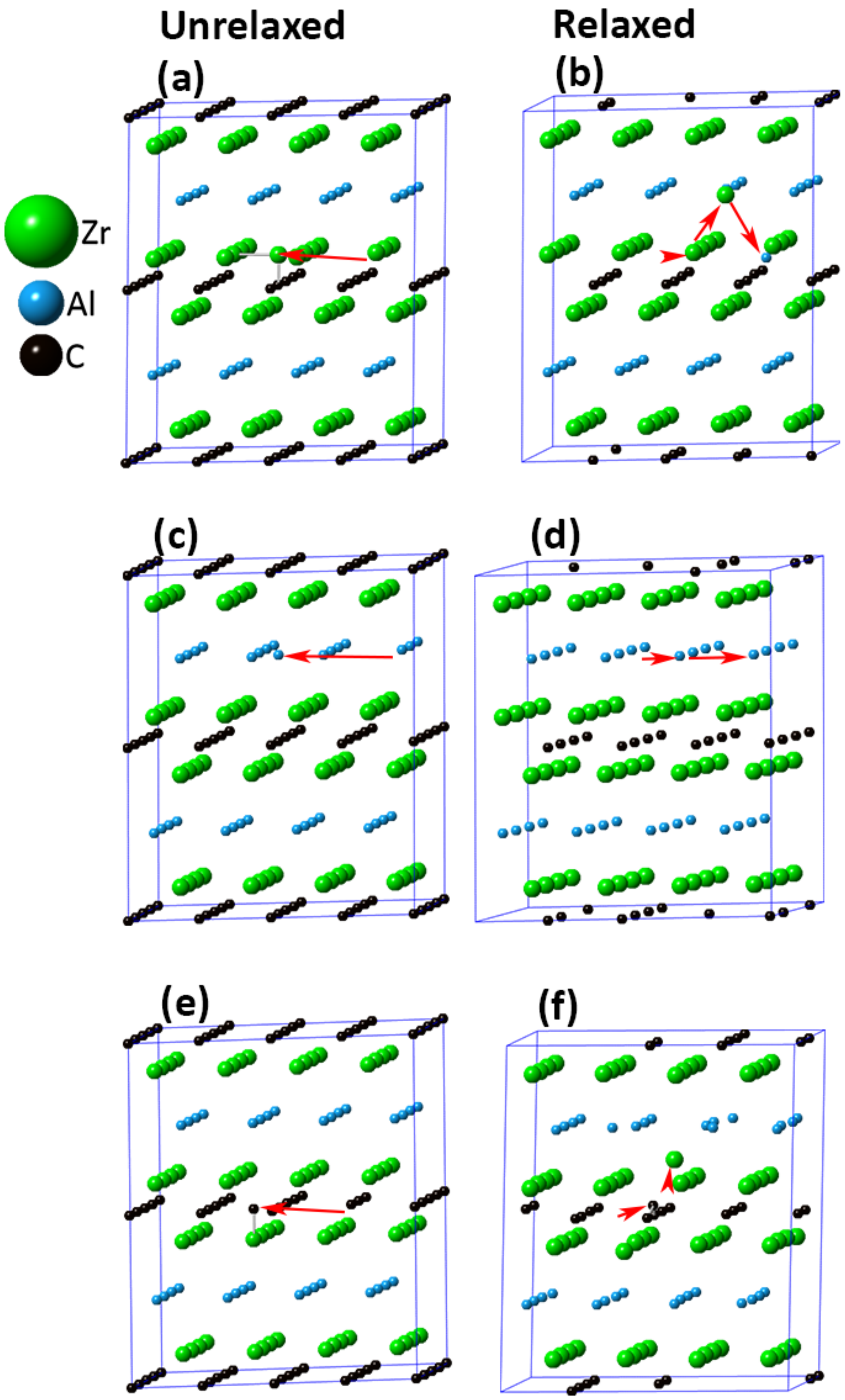

Figure S13: The unrelaxed and relaxed structures of Zr_2AlC with different types of self-Frenkel pairs. (a) Unrelaxed V_{Zr}+Zr_i (b) Relaxed V_{Zr}+Zr_i (c) Unrelaxed V_{Al}+ Al_i (d) Relaxed V_{Al}+Al_i (e) Unrelaxed V_C+C_i (f) Relaxed V_C+C_i. Arrows indicate how atoms are displaced initially and then during relaxation.

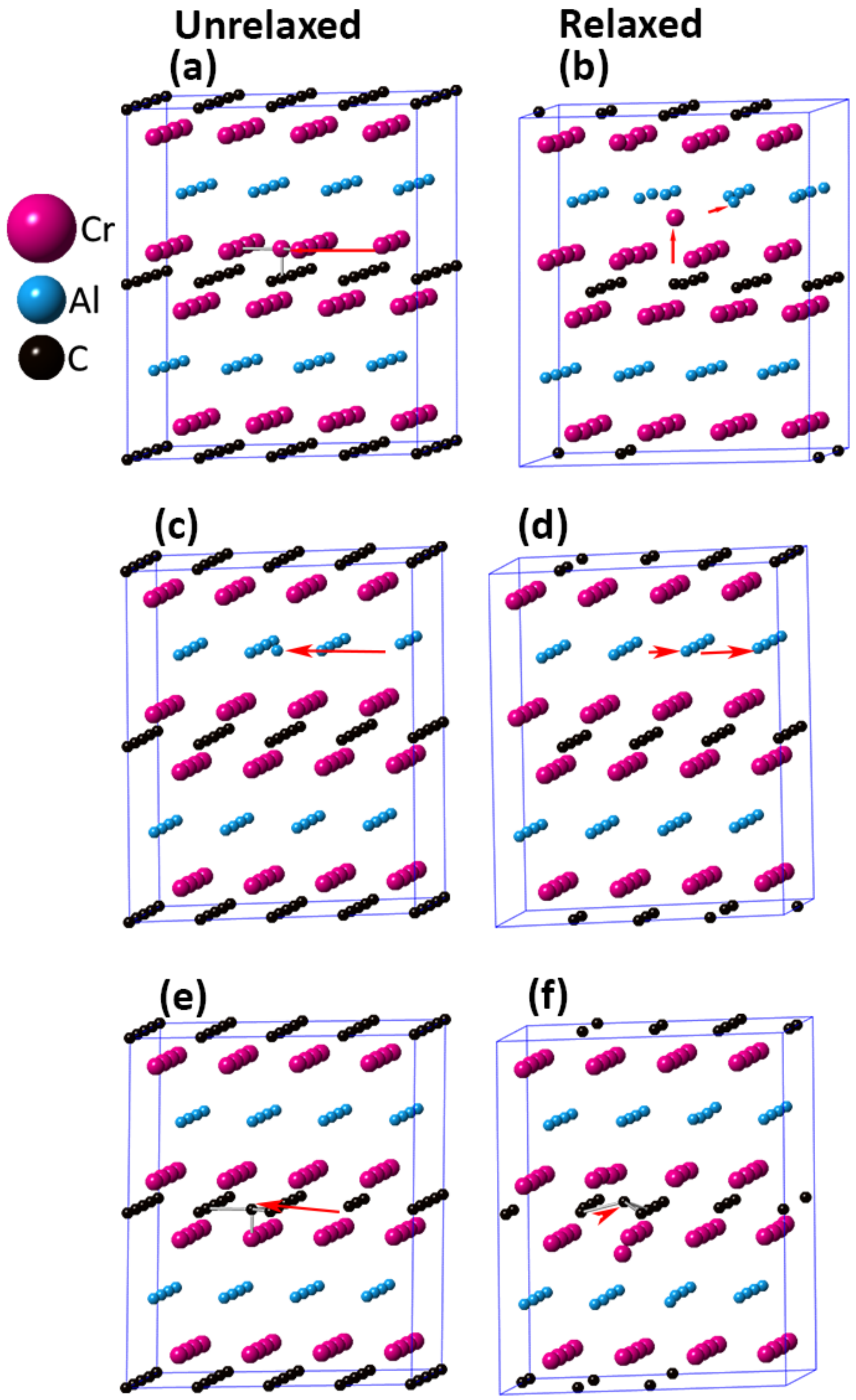

Figure S14: The unrelaxed and relaxed structures of Cr_2AlC with different types of self-Frenkel pairs. (a) Unrelaxed V_{Cr}+Cr_i (b) Relaxed V_{Cr}+Cr_i (c) Unrelaxed V_{Al}+ Al_i (d) Relaxed V_{Al}+Al_i (e) Unrelaxed V_C+C_i (f) Relaxed V_C+C_i. Arrows indicate how atoms are displaced initially and then during relaxation.

Calculation of the Bader charges and bond strengths using QTAIMAC

According to QTAIMAC, a crystal can be subdivided into disjoint regions called atomic basins by defining interatomic surfaces of electron density, $\rho(r)$, which satisfy a zero-flux condition i.e., $\nabla\rho(r).n(r) = 0$ where *n(r)* is a vector normal to the interatomic surface at position *r*. Once the atomic basins are defined, atomic properties such as Bader charges can be calculated by integrating the charge density inside each atomic basin. The charge density topology can be determined with the aid of four special points called critical points (CP) of charge density which are solutions of $\nabla\rho(r) = 0$. The maxima and minima of electron density are called the nuclear critical point (ncp) and cage critical point (ccp) respectively. The first and second order saddle points of electron density are called the bond critical point (bcp) and ring critical point (rcp) respectively. The bond critical point (bcp) lies on a bond path that defines a bond between two atoms. The charge density and its Laplacian at the bcp describe the strength and nature of the bond respectively. High charge density at a bcp indicates strong interatomic bonding. A positive Laplacian of charge density at a bcp indicates a closed shell interaction (ionic) and a negative value of the Laplacian indicates a shared shell interaction (covalent). Bonding in MAX phases is more complicated; therefore, the sign of the Laplacian of charge density at a bond critical point is only indicative of the more dominant bonding type.

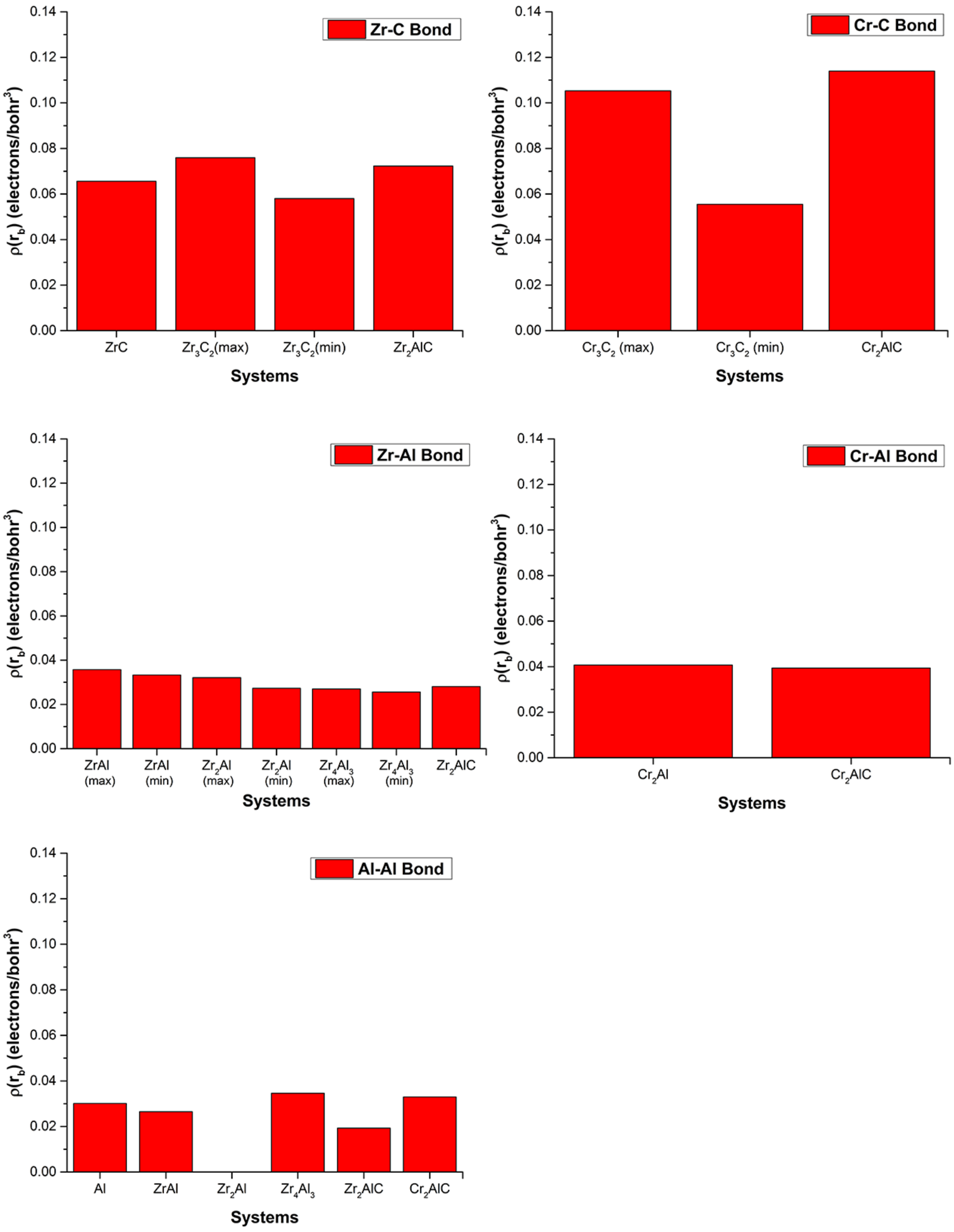

Figure S15: Charge densities at bond critical points for M-C, M-Al and Al-Al (M=Zr/Cr) bonds in Zr_2AlC, Cr_2AlC and the relevant binary compounds and metallic systems.

References:

1. Lapauw, T., et al., *Synthesis of the new MAX phase Zr_2AlC.* Journal of the European Ceramic Society, 2016. **36**(8): p. 1847-1853.
2. Manoun, B., et al., *Compression behavior of M_2AlC (M = Ti, V, Cr, Nb, and Ta) phases to above 50GPa.* Physical Review B, 2006. **73**(2): p. 024110.
3. Huang, Q., et al., *Saturation of ion irradiation effects in MAX phase Cr_2AlC.* Acta Materialia, 2016. **110**: p. 1-7.
4. Han, H., et al., *A first-principles study on the defective properties of MAX phase Cr_2AlC: the magnetic ordering and strong correlation effect.* RSC Advances, 2016. **6**(87): p. 84262-84268.
5. Xiao, J., et al., *Investigations on Radiation Tolerance of $M_{n+1}AX_n$ Phases: Study of Ti_3SiC_2, Ti_3AlC_2, Cr_2AlC, Cr_2GeC, Ti_2AlC, and Ti_2AlN.* Journal of the American Ceramic Society, 2015. **98**(4): p. 1323-1331.
6. Yembadi, R. and B.B. Panigrahi, *Thermodynamic Assessments and mechanically activated synthesis of ultrafine Cr_2AlC MAX phase powders.* Advanced Powder Technology.
7. Buckeridge, J., et al., *Automated procedure to determine the thermodynamic stability of a material and the range of chemical potentials necessary for its formation relative to competing phases and compounds.* Computer Physics Communications, 2014. **185**(1): p. 330-338.
